# Hypouricemic Effect of 2,5-Dihydroxyacetophenone, a Computational Screened Bioactive Compound from *Ganoderma applanatum*, on Hyperuricemic Mice

**DOI:** 10.3390/ijms19051394

**Published:** 2018-05-07

**Authors:** Danling Liang, Tianqiao Yong, Shaodan Chen, Yizhen Xie, Diling Chen, Xinxin Zhou, Dan Li, Muxia Li, Lu Su, Dan Zuo

**Affiliations:** 1 School of Pharmaceutical Science, Guangzhou University of Chinese Medicine, Guangzhou 510006, China; liangdanling@hotmail.com (D.Liang); 13751717863@163.com (D.Li); 13760722769@163.com (M.L.); 2 Guangdong Yuewei Edible Fungi Technology Co., Guangzhou 510663, China; chenshaodan@126.com (S.C.); 13622216490@126.com (Y.X.); diling1983@163.com (D.C.); sulu5921@163.com (L.S.); 3 State Key Laboratory of Applied Microbiology Southern China, Guangdong Provincial Key Laboratory of Microbial Culture Collection and Application and Guangdong Open Laboratory of Applied Microbiology, Guangdong Institute of Microbiology, Guangzhou 510070, China; 4 Guangzhou Institutes of Biomedicine and Health, Chinese Academy of Sciences, Guangzhou 510530, China; zuo_dan@gibh.ac.cn

**Keywords:** hyperuricemia, 2,5-dihydroxyacetophenone, *Ganoderma applanatum*, bioactive compound

## Abstract

Searching novel hypouricemic agents of high efficacy and safety has attracted a great attention. Previously, we reported the hypouricemic effect of *Ganoderma applanatum*, but its bioactives, was not referred. Herein, we report the hypouricemic effect of 2,5-dihydroxyacetophenone (DHAP), a compound screened from *Ganoderma applanatum* computationally. Serum parameters, such as uric acid (SUA), xanthine oxidase (XOD) activity, blood urea nitrogen (BUN), and creatinine were recorded. Real-time reverse transcription PCR (RT-PCR) and Western blot were exploited to assay RNA and protein expressions of organic anion transporter 1 (OAT1), glucose transporter 9 (GLUT9), uric acid transporter 1 (URAT1), and gastrointestinal concentrative nucleoside transporter 2 (CNT2). DHAP at 20, 40, and 80 mg/kg exerted excellent hypouricemic action on hyperuricemic mice, reducing SUA from hyperuricemic control (407 ± 31 μmol/L, *p* < 0.01) to 180 ± 29, 144 ± 13, and 139 ± 31 μmol/L, respectively. In contrast to the renal toxic allopurinol, DHAP showed some kidney-protective effects. Moreover, its suppression on XOD activity, in vivo and in vitro, suggested that XOD inhibition may be a mechanism for its hypouricemic effect. Given this, its binding mode to XOD was explored by molecular docking and revealed that three hydrogen bonds may play key roles in its binding and orientation. It upregulated OAT1 and downregulated GLUT9, URAT1, and CNT2 too. In summary, its hypouricemic effect may be mediated by regulation of XOD, OAT1, GLUT9, URAT1, and CNT2.

## 1. Introduction

Uric acid, as a sparingly soluble compound, is the end product of purine catabolism in humans, since the silent mutation of uricase in human evolution. It is accumulated in tissues and extracellular fluid in the appearance of monosodium urate (MSU) [1]. When the serum uric acid (SUA) concentration exceeds 6.8 mg/dL for men and 6.0 mg/dL for women, it is defined as hyperuricemia (HUA) [2]. Long-term HUA may cause deposition of MSU in the joints, and then trigger repeated episodes of inflammation, clinically defined as gout [3]. Disarrangement of purine metabolism or uric acid excretion may contribute HUA. With the intake of high purine diets, HUA has become a high prevalent disease [4]. Many lines of evidence suggest that HUA is associated with a variety of chronic metabolic diseases, such as cardiovascular disease, obesity, and renal diseases [5,6,7].

**Figure 1:**
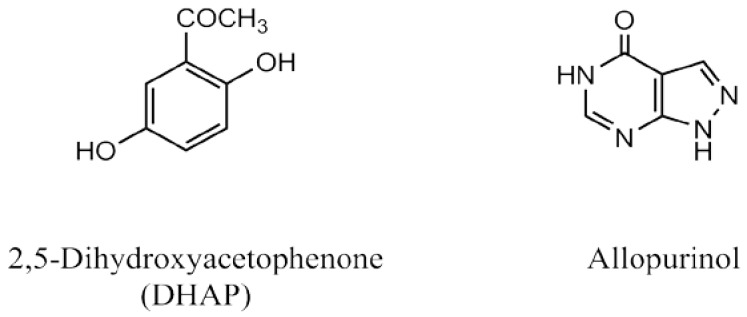
Structures of DHAP and allopurinol.

At present, clinical medicines against HUA can be classified into three categories: (i) xanthine oxidase (XOD) inhibitors, such as allopurinol or febuxostat, decreasing the production of uric acid through metabolism; (ii) uricosuric agents, such as benzbromarone and probenecid, elevating uric acid excretion by interacting with renal uric acid transporters; and (iii) uricase analogues, such as pegloticase, metabolizing uric acid to dissolvable allantoin [8,9]. However, they exert some fatal adverse effects [3]. Therefore, novel and non- or low-toxic agents are highly sought after. Several studies have focused on natural products from traditional Chinese medicine that remove dampness by promoting diuresis, because of their long-term efficacy and safety against HUA [10,11,12]. *Ganoderma applanatum* was recorded as a diuresis agent [12] in Chinese herbal classic literature, and closely associated with the prevention of HUA. Clinically, *G. applanatum* as a folk medicine, has been exploited to prevent and treat various diseases since 100 BC, such as hypertension, diabetes, hepatitis, cancers, and acquired immune deficiency syndrome (AIDS) [13]. Various bioactives, such as polysaccharides, sterols, and triterpenes [14,15], presented broad pharmacologic activities, including antimicrobial, antioxidant, antitumor, immunostimulating regulation, and hepatoprotective activities [15,16,17,18]. Previously, we reported that *G. applanatum* resisted HUA through enhancing uric acid excretion by downregulating GLUT9 (glucose transporter 9) and upregulating OAT1 (organic anion transporter 1) and exhibited some nephron- and liver-protective effects [19]. Nonetheless, the bioactive mechanism against HUA is not yet clear. Given that, computational virtual screening was performed using the XOD structure, and 2,5-dihydroxyacetophenone (DHAP, Figure 1) ranked high [20].

In this paper, a systematic study was launched to investigate the hypouricemic effect of DHAP. Firstly, XOD inhibitory effect of DHAP was assayed, in vitro, to verify the prediction. Then, its hypouricemic effects were examined on hyperuricemic mice, wherein, SUA, BUN (blood urea nitrogen) and creatinine were recorded. RNA and protein expressions of OAT1, GLUT9, URAT1 (uric acid transporter 1), and CNT2 (gastrointestinal concentrative nucleoside transporter 2) were examined by RT-PCR and Western blot. Also, XOD activities, in vitro, in serum, were tested by enzyme-linked immunosorbent assay (ELISA) method. Due to its inhibition against XOD, molecular docking was conducted to explore the details of the binding of DHAP to XOD.

## 2. Results

### 2.1. In Vitro Enzyme Activity Assay

**Figure 2:**
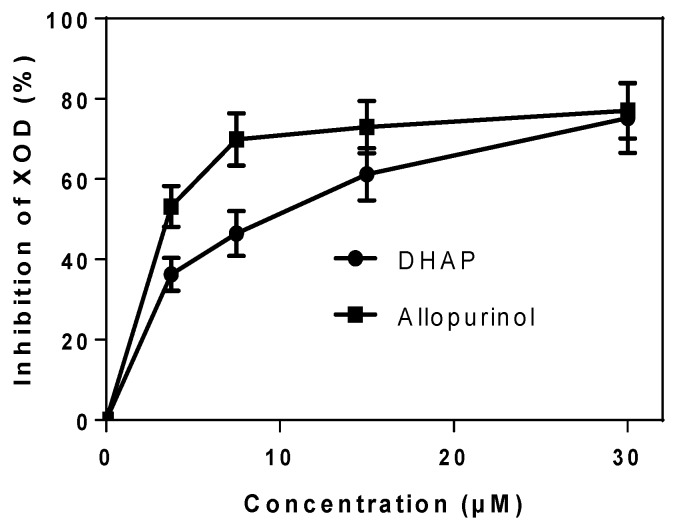
Xanthine oxidase (XOD) inhibition by DHAP. Phosphate buffer saline used as negative control.

To determine the suppressive effects of DHAP against XOD, we performed the XOD inhibitory assay with phosphate buffer saline (PBS) and allopurinol as negative and positive controls, respectively. As shown in Figure 2, the activity of XOD was inhibited by DHAP in a concentration-dependent manner. The IC_50_ (8.12 ± 0.27 μM) was higher than allopurinol (2.22 ± 0.21 μM). The results indicated that DHAP had the ability to inhibit XOD in vitro, implying a hypouricemic effect.

### 2.2. Animal Experiment

**Figure 3:**
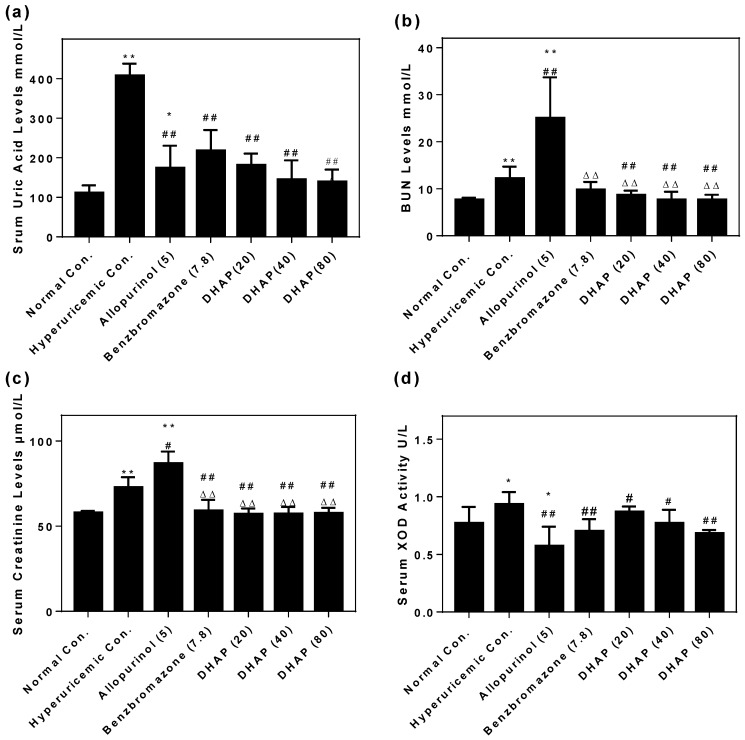
Effects of DHAP on serum uric acid (**a**); BUN (**b**); creatinine (**c**); and XOD activity (**d**). *n* = 8. * *p* < 0.05, ** *p* < 0.01 versus the normal control; ^#^ *p* < 0.05, ^##^ *p* < 0.01versus hyperuricemic control; ^△△^ *p* < 0.01 compared with allopurinol control.

To verify its hypouricemic effect in vivo, we performed an animal experiment using hyperuricemic mice. Figure 3a depicted the notable increase of the SUA of the HUA control (407 ± 31 μmol/L, *p* < 0.01) when compared to normal mice (111 ± 20 μmol/L), confirming that the model was established successfully. The oral treatment with allopurinol at 5 mg/kg and benzbromarone at 7.8 mg/kg as positive controls elicited significantly reductions in SUA to 173 ± 56 and 217 ± 52 μmol/L in hyperuricemic mice (*p* < 0.01). It was interesting that DHAP at 20, 40, and 80 mg/kg decreased SUA of hyperuricemic mice to 180 ± 29, 144 ± 13, and 139 ± 31 μmol/L (*p* < 0.01), demonstrating a significant hypouricemic effect.

To illuminate its impact on renal function, the related parameters were assayed (Figure 3b). The obvious increase of BUN in hyperuricemic control (12.14 ± 2.56 mmol/L) in comparison to normal mice (7.61 ± 0.48 mmol/L, *p* < 0.01) illustrated some adverse influence on renal function by the high dose of PO (potassium oxonate). Allopurinol (24.97 ± 8.70 mmol/L, *p* < 0.01) further elevated BUN, indicating serious impairment of renal function, whereas, benzbromarone (9.75 ± 1.71 mmol/L) and DHAP (8.58 ± 1.00, 7.60 ± 1.76 and 7.62 ± 1.11 mmol/L) at 20, 40, and 80 mg/kg exhibited notable decreases in BUN, in contrast with the hyperuricemic and allopurinol controls, respectively (*p* < 0.01).

In addition, the hyperuricemic control (72.57 ± 6.14 μmol/L, *p* < 0.01) elevated the serum creatinine as compared to the normal control (57.72 ± 1.19 μmol/L, Figure 3c). Allopurinol (86.73 ± 7.06 mmol/L, *p* < 0.01) increased the serum creatinine further, demonstrating some damage on renal function. However, benzbromarone and DHAP with various doses presented serum creatinine at 58.86 ± 6.58, 56.99 ± 3.37, 57.08 ± 4.16, and 57.49 ± 3.21 μmol/L, which were similar to the normal control. Therefore, DHAP may protect the renal function, which was consistent with BUN analysis.

To examine the effect of DHAP on XOD in vitro, serum XOD activities were tested. Figure 3d showed a slight increase of hyperuricemic control (0.903 ± 0.108 U/L) in comparison with the normal control (0.767 ± 0.143 U/L). Allopurinol lowered the serum XOD activity (0.570 ± 0.169 U/L, *p* < 0.01) in hyperuricemic mice since it’s a XOD inhibitor clinically. DHAP also demonstrated suppressive effects on XOD (0.866 ± 0.049, 0.768 ± 0.118, and 0.679 ± 0.032U/L, *p* < 0.05 or 0.01).

**Figure 4:**
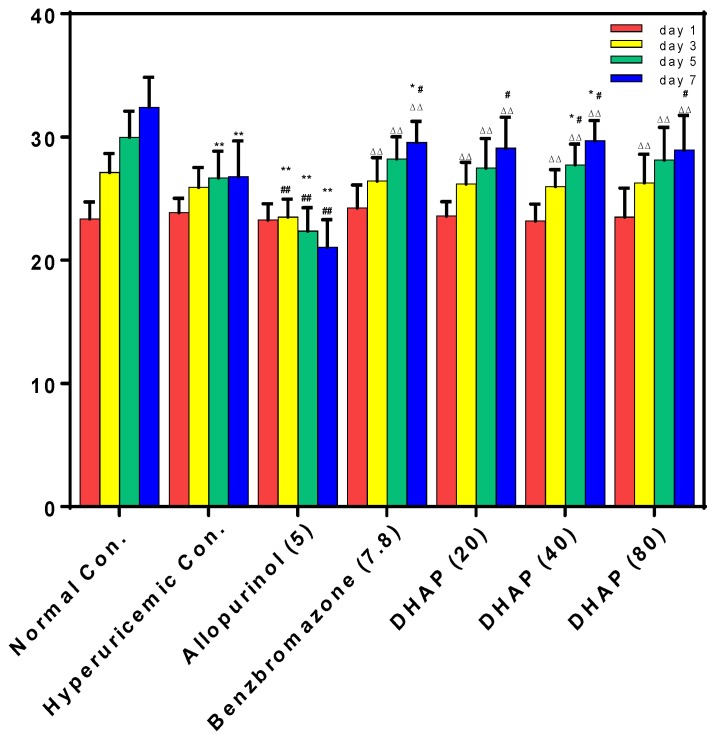
Body weight. *n* = 8. * *p* < 0.05, ** *p* < 0.01 versus the normal control; ^#^ *p* < 0.05, ^##^ *p* < 0.01 versus hyperuricemic control; ^△△^ *p* < 0.01 compared with allopurinol control.

Hyperuricemic control presented a significant reduction (26.68 ± 2.17 and 26.78 ± 2.90 g, *p* < 0.01) in body weight on the 5th and 7th day compared to the normal control (29.98 ± 2.13 and 32.41 ± 2.43 g, Figure 4) because of the administration of PO at high dose. Furthermore, allopurinol decreased body weights because of its serious toxicity. Benzbromarone and DHAP at various doses had little impact on body weight compared to normal control.

**Figure 5:**
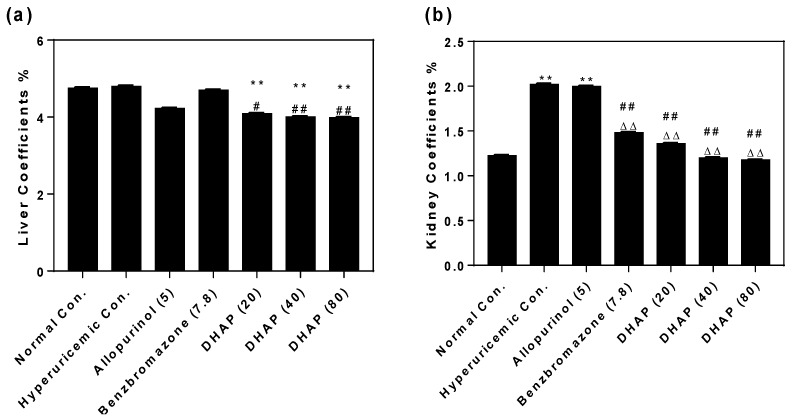

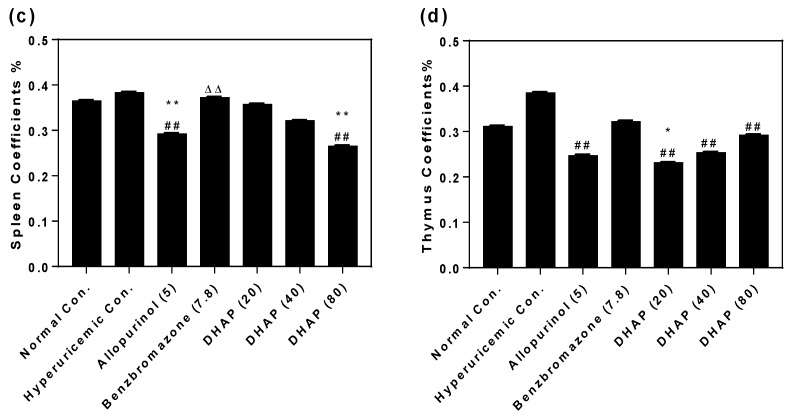
Coefficients of liver (**a**); kidney (**b**); spleen (**c**) and thymus (**d**). *n* = 8. * *p* < 0.05, ** *p* < 0.01 versus the normal control; ^#^ *p* < 0.05, ^##^ *p* < 0.01versus hyperuricemic control; ^△△^ *p* < 0.01 compared with allopurinol control.

To further assess its influence on inner organs, organ coefficients were recorded. Normal control, hyperuricemic control, and benzbromarone control had no significant difference in liver coefficients (Figure 5a). However, liver coefficients were decreased by allopurinol and DHAP, which may be related to some liver toxicity. Renal coefficients of hyperuricemic (2.02%) and allopurinol (2.00%) controls were significantly higher than that of the normal group (1.23%, *p* < 0.01), indicating nephrotoxicity of PO and allopurinol (Figure 5b). Renal coefficients of benzbromarone control and DHAP groups (1.48%, 1.36%, 1.20% and 1.18%) did not show significant differences compared with the normal group. The spleen and thymus coefficients both showed slight elevations in the hyperuricemic control (Figure 5c,d), which may be associated with hyperuricemia-induced inflammatory. There were clear decreases of thymus coefficients of allopurinol control and DHAP groups. Importantly, thymus coefficients of DHAP groups (0.23%, 0.25%, and 0.29%) become closer to the normal group (0.31%).

**Figure 6:**
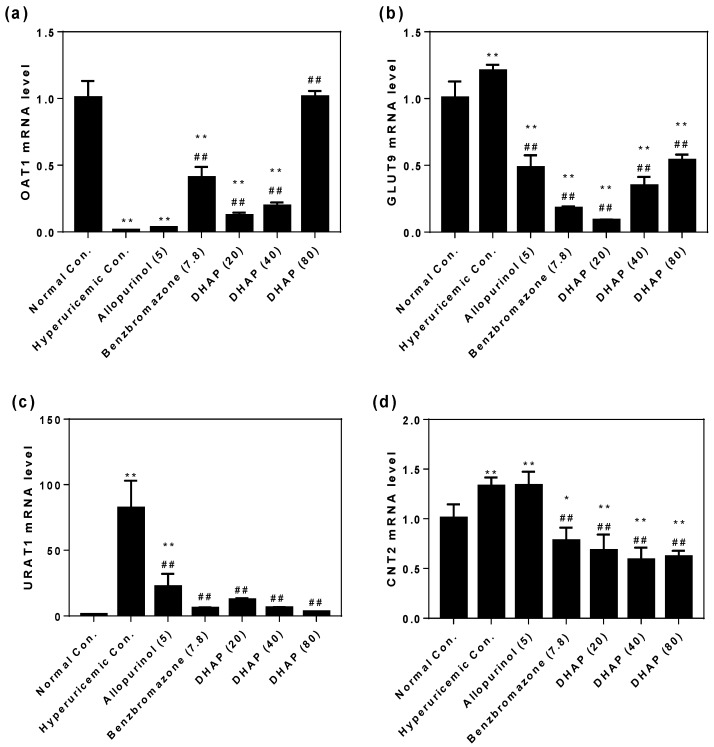
Effects of DHAP on renal OAT1 (**a**); GLUT9 (**b**); URAT1 (**c**); and intestinal CNT2 (**d**) mRNA expressions. *n* = 3. * *p* < 0.05, ** *p* < 0.01 versus the normal control; ^##^ *p* < 0.01 versus hyperuricemic control.

To date, several studies have revealed that renal transporters for uric acid secretion and resorption [21], and gut intestinal transporters for purine nucleoside absorption played key roles in hyperuricemia [22]. Therefore, the effects of DHAP on them were examined. Potassium oxonate (PO) and hypoxanthine (HX) significantly reduced the mRNA expression of OAT1 in hyperuricemic control (*p* < 0.01, Figure 6a) in comparison with normal control. DHAP and benzbromarone elevated OAT1 mRNA expression in hyperuricemic mice (*p* < 0.01). The mRNA of OAT1 in high-dosed DHAP group was similar to normal mice, which may imply its restoration of the uric acid excretion. PO and HX elevated GLUT9, URAT1, and CNT2 mRNAs (*p* < 0.01, Figure 6b–d) in hyperuricemic models. The mRNA increases of GLUT9, URAT1, and CNT2 were apparently reversed by benzbromarone and DHAP at various doses (*p* < 0.01) in hyperuricemic mice. Particularly, URAT1 and CNT2 mRNAs were downregulated in dose-dependent manner, indicating that DHAP may suppress uric acid reabsorption and purine nucleoside absorption.

**Figure 7:**
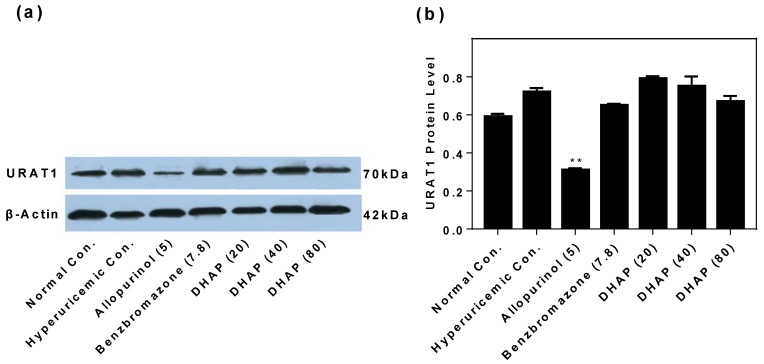
Effects of DHAP on renal URAT1 protein by Western blot analysis: (**a**) immunoreactive bands and (**b**) densitometries normalized (expressed as mean ± SD; *n* = 3). ** *p* < 0.01 versus the normal control.

Also, URAT1 protein was accessed by Western blot, which revealed that DHAP at high doses caused downregulation (Figure 7).

### 2.3. Computational Studies

**Figure 8:**
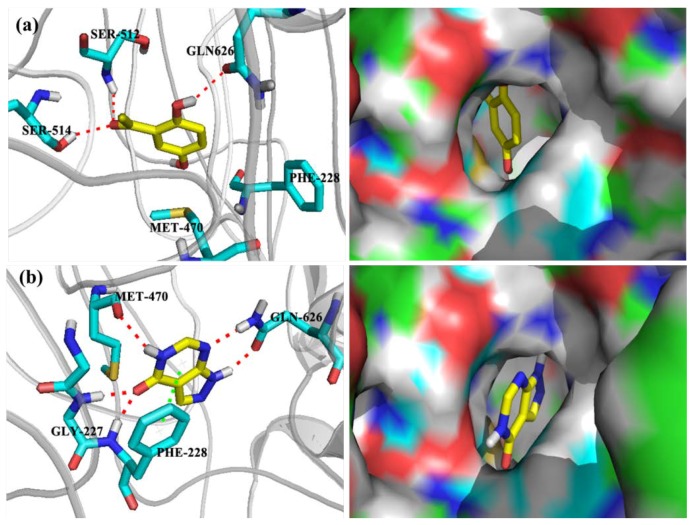
Binding modes of DHAP (**a**) and allopurinol (**b**) to XOD. The red dashed lines represent hydrogen bond left; the green dashed lines the π–π stacking left; green color carbon atoms right; red the oxygen atoms right; blue the nitrogen atoms right.

To investigate the possible binding mode of DHAP to XOD, molecular docking was performed (Figure 8). The docking score, glide score, along with glide emodel of DHAP and allopurinol to XOD were listed in Table 1. In terms of DHAP, it interacted with XOD through three hydrogen bonds. Specifically, the hydrogen atoms of the amino group of SER512 and the hydroxyl group of SER514 formed hydrogen bonds to the oxygen atom of the carbonyl group of DHAP (Figure 8a). Besides, another hydrogen bond was generated by oxygen atom of amide group of GLN626 and hydrogen atom of the hydroxyl group attached to DHAP. In contrast, there were five hydrogen bonds and a Pi–Pi stacking between allopurinol and XOD (Figure 8b). Specifically, the oxygen atom of carbonyl group in allopurinol produced two hydrogen bonds to hydrogen atoms of two amide groups in GLY227. Regarding to GLN626, oxygen atom and hydrogen atom of the amide group bonded via two hydrogen bonds to hydrogen atom attached to the five-membered *N*-heterocycle and nitrogen atom in six-membered *N*-heterocycle in allopurinol, respectively. Hydrogen atom attached on amide of the six-membered ring also formed a hydrogen bond to the oxygen atom of MET470. Moreover, the aromatic six-member heterocyclic ring of allopurinol was involved in π–π stacking to the phenyl ring on PHE228. Commonly, DHAP and allopurinol formed hydrogen bonds with GLN626, and high affinities were observed. However, the specific binding modes are different, where allopurinol owned more interactions with XOD and had higher docking scores than DHAP.

## 3. Discussion

In this study, we examined the hypouricemic action of DHAP using XOD activity assay in vitro, hyperuricemic mouse models in vivo, and molecular docking analysis in silico. DHAP did show an excellent hypouricemic effect through inhibiting XOD, suppressing URAT1, GLUT9, and CNT2, and elevating OAT1, which was comparable to allopurinol and benzbromarone. Renal injuries of hyperuricemic mice were alleviated by DHAP. Also, anti-inflammatory effects and little toxicity were observed.

DHAP is known to induce cell death through inducing apoptosis via regulating the Ras–mitogen-activated protein kinase (MAPK) activation pathway [23]. Moreover, an anti-inflammatory effect was also observed for DHAP [24]. This makes it a potentially good candidate against cancer and inflammation. Importantly, it was predicted to be a bioactive compound against hyperuricemia through suppressing XOD. To determine DHAP’s hypouricemic action in vitro, enzymatic activity of XOD in its presence was tested, and it showed a significant inhibitory action, implying a hypouricemic effect. Hyperuricemic animal models are the key for hyperuricemia-related disease research. HX and PO are conventional agents for model establishment. Herein, large amounts of PO, together with HX, promoted SUA remarkably, and some renal injuries and inflammatory [25] were observed for the hyperuricemic control, mimicking human hyperuricemia. Using this model, the hypouricemic effects of DHAP was examined. DHAP showed an excellent hyperuricemic effect and some protective impacts on renal function, which reduced SUA, serum creatinine, and BUN.

DHAP, as a pure compound, maybe trigger skin and eye irritation, as well as respiratory irritation [26]. It is known that some pure organic compound drugs cause some side reactions against skin, eyes, or the respiratory system. However, these drawbacks did not prevent them from being used as drugs under specific usage conditions. For example, allopurinol is used as a classic drug against hyperuricemia, although it is toxic if swallowed, and may cause some allergic skin reactions [27], or even the fatal Stevens–Johnson syndrome. However, toxicity examination is important for all drug or food research. In toxicological research, comparison of body and inner organ weights has been traditionally used to evaluate toxic action. In this study, PO and HX suppressed weight growth. However, DHAP reversed it, showing some toxicity alleviation effect. However, allopurinol and DHAP decreased liver coefficients, demonstrating some negative impacts on liver function, which was consistent with the point that they both targeted XOD, which was primarily present in liver. On the other hand, DHAP decreased the renal coefficients elevated by PO, demonstrating that it may alleviate the negative impact of PO on renal function. This is consistent with the results of BUN and creatinine. DHAP relieved spleen and thymus coefficients increased by the inflammatory effects. It is known that HUA is a disease accompanied by inflammation induced by the deposition of MSU [28]. Interestingly, thymus coefficients showed an positive correlation to the doses of DHAP, while spleen coefficients showed a negative correlation. Further studies, which take these into account, will need to be undertaken.

XOD is a key target for hyperuricemia, since it is the key enzyme for uric acid production via purine metabolism. Sometimes, hyperuricemic patient show improved XOD activity [29]. Since DHAP entered firstly into blood in the treatment process, it may bond to serum XOD. Therefore, we mainly assayed serum XOD activities. Serum XOD activities were reduced in vivo, which is consistent with the result in vitro. Hence, DHAP possessed uric acid-lowering activity in vivo, and inhibited xanthine oxidase activity in vitro. Thus, its interaction with XOD was validated. Besides, bovine XOD structure (for example, PDBID: 1FIQ) is a classic structure, and has been frequently used for hypouricemic candidate docking or screening [30]. Therefore, it was selected as a target to be docked by DHAP to explore insights into the observed activities and elucidate the binding mode. The three hydrogen bonds involved in binding might play a key role for orientation and location in active site.

Hyperuricemic patients raised by poor uric acid excretion since their renal dysfunction are more than 90%, clinically [31]. OAT1, GLUT9, and URAT1 have key roles in uric acid excretion. OAT1 functions in uric acid secretion initially [32], while GLUT9 [33] and URAT1 [34] have roles in uric acid reabsorption. CNT2 is responsible for the absorption of purine nucleosides in the gastrointestinal tract [22]. Their expression was examined, and results demonstrated that the upregulation of OAT1 and downregulation of GLUT9, URAT1, as well as CNT2, might be attributed to the hypouricemic action of DHAP.

Due to its excellent hypouricemic effect in vivo and vitro, use of DHAP as a template to design and synthesize analogs with improved pharmacological properties against hyperuricemia or even metabolic syndrome should be underway. This research provided evidence that DHAP was a promising hypouricemic agent, which may associate with several pathways. Further research should be focused on its structure modification and optimization.

## 4. Materials and Methods 

### 4.1. Materials

XOD (0.6 units/mg) from bovine milk was purchased from Sigma-Aldrich LLC. (St. Louis, MO, USA). Xanthine (99.5%), PO (98.0%), HX (99%), DHAP (98%), and benzbromarone (98%) were acquired from Aladdin Reagent Co. (Shanghai, China). Allopurinol (98%) was obtained from Tokyo Chemical Industry Co. (Tokyo, Japan). Assay kits for uric acid, blood urea nitrogen, and creatinine levels were supplied by Mindray Medical Corp. (Shenzhen, China). Assay kits for XOD activity were bought from R&D System Inc. (Minneapolis, MN, USA). TRIZOL reagent and PCR primers were acquired from Servicebio (Wuhan, China).

### 4.2. In Vitro XOD Inhibition Assay

The XOD inhibitory assaying was performed based on the report by Nguyen et al. [35] with slight modifications. The stock solutions of XOD (0.05 units/mL), xanthine (0.5 mM), DHAP, and allopurinol in a series of various amounts (3.75, 7.5, 15, 30 μM) were prepared by dissolving with 70 mM phosphate buffer solution (PBS, pH 7.5) freshly. After incubation at 37 °C of 40 μL XOD and 40 μL sample solutions for 30 min, the reaction was initiated by the addition of 120 μL of xanthine solution. The absorbance at 290 nm was monitored using Infinite^®^ 200 Pro NanoQuant multimode microplate reader (Tecan Group Ltd., Männedorf, Switzerland) every 1 min up to 8 min. Allopurinol and PBS were used as positive and negative controls, respectively. Three replicates were repeated for each. The inhibition was calculated as Equation (1):

Inhibition % = (1 − B/A) × 100(1)

where A and B are the slopes of the reaction with and without sample.

### 4.3. Animals

All animal experimental protocols used in this study were approved by Guangdong Institute of Microbiology (GT-IACUC20171109-1, 9 November 2017, Guangzhou, China). Male-specific pathogen-free (SPF) Kunming mice (20 ± 2 g) were supplied by the Guangdong Provincial Medical Laboratory Animal Centre (Guangzhou, China). All mice were housed in laboratory conditions for 1 week before the experiment. Mice were allowed to have food and water freely. Temperature was maintained between 24 to 26 °C. In the preliminary experiments, mice were randomized into several groups (*n* = 10): normal control, hyperuricemic control, allopurinol control, benzbromarone control, and drug groups, with DHAP at doses of 20, 40, and 80 mg/kg respectively. The hypouricemic mice models were established by a method reported by us [36], with an increased usage of PO. Allopurinol and benzbromarone were used as positive controls. Hyperuricemic mice were injected intraperitoneally with PO at a dose of 300 mg/kg, and pre-treated orally with HX at a dose of 500 mg/kg, to increase the SUA level, 1 h before the drug administration. Mice used as normal controls were injected intraperitoneally and administrated orally with the same volume of physiological saline (0.9%) as the hyperuricemic models.

### 4.4. Drug Administration

Prior to drug administration, mice were fasted for 1 h, but given water ad libitum. Mice in each treatment were administered once a day for a week. Among them, mice of normal and hyperuricemic control were only administered orally with the same volume of physiological saline (0.9%). Mice of allopurinol and benzbromarone controls were administered intragastrically with allopurinol (5 mg/kg) and benzbromarone (7.8 mg/kg). For drug groups, mice were administrated orally with DHAP at the doses of 20, 40, 80 mg/kg, respectively.

### 4.5. Mensuration of Uric Acid Level, XOD Activities, BUN, and Creatinine Levels

All mice were sacrificed after 7 days of treatment. The whole blood samples were collected for separation of the serum at 3800 rpm for 10 min at 4 °C, and stored at −20 °C. The serum was applied for determination of uric acid, XOD activity, blood urine nitrogen (BUN), and creatinine. Liver, renal, thymus, and small intestine tissues were excised, weighted, and homogenized with cold physiological saline (0.9%), and centrifuged at 2400 rpm for 10 min at 4 °C. The supernatants were deserved for XOD activity test. XOD activities were determined by a colorimetric method using ELISA kits. Specially, SUA, BUN, and serum creatinine were measured by exploiting BS-480 Mindray Automatic Clinical Blood Chemistry Analyzer (Mindray Medical Corp., Shenzhen, China).

### 4.6. Organ Coefficients

Organ coefficients, expressed as tissue weighting factor, were calculated by dividing the weight of organ by the corresponding weight of individual mouse.

### 4.7. Statistical Analysis

The statistical analysis was carried out using the professional data-processing program SPSS (Release 17.0, SPSS Inc., Chicago, IL, USA, 2001). All data was expressed as mean ± standard deviation (SD), and analyzed by one-way analysis of variance (ANOVA). A difference was considered significant at the *p* < 0.05 or *p* < 0.01 level.

### 4.8. RT-PCR Analysis of URAT1, GLUT9, OAT1, and CNT2

Total RNA extractions were performed using TRIzol reagent. After homogenization of the kidney and small intestine tissues, the obtained liquid was mixed with chloroform and centrifuged, followed by precipitating the aqueous phase with an equivalent volume of isopropanol. After washing with ethanol (75%), the total RNA pellets were suspended using diethylpyrocarbonate (DEPC) water. Total RNA (2 μg) was added to each of the tubes, together with 4 μL of 5× reaction buffer, 2 μL dNTPs (10 mmol/L), 1 μL of oligo (dT) 18, 1 μL of RNA inhibitor, and 1 μL of M-MLV reverse transcriptase (200 U/μL), and the tube volumes were adjusted to 20 μL using DEPC water without RNase. The tubes were kept at 42 °C for 60 min, and then the reactions were stopped by heating the RNA solution at 70 °C for 5 min. The obtained cDNA was diluted with DNase-free water, and PCR amplification was performed using primers at appropriate conditions (Table 2). Tubes containing 12.5 μL of 2× qPCR Mix (Servicebio, Wuhan, China), 2.0 μL of 7.5 μmol·L^−1^ gene primers, and 2.5 μL of cDNA, were adjusted to a volume of 20 μL with DEPC-treated water. PCR was performed with an initial heat denaturation at 95 °C for 10 min, and the PCR cycles were repeated 40 times under the following conditions: denaturation at 95 °C for 15 s, annealing at 60 °C for 60 s, and extension at 95 °C for 20 s, and the last extension at 72 °C for 5 min. GAPDH was used as an endogenous standard. Finally, the PCR products were quantified by electrophoresis.

### 4.9. Western Blot Analysis

Kidney samples were homogenized with 10 equivalent volumes of RIPA lysis buffer, supplemented with 1 mM PMSF (protease inhibitor), in an ice bath for 30 min, and then centrifuged (12,000× *g*, 10 min) to afford total proteins, followed by determining concentration by BCA Protein Assay Kit (Tiangen Biotech Co., Beijing, China). An equivalent of 5 μL protein samples were separated by 10% SDS-PAGE and then transferred onto PVDF membrane (Millipore, Burlington, MA, USA). The non-specific binding sites of the membranes were blocked with 5% skimmed milk in TBST (Tris-buffered saline with 0.1% Tween-20). Then, the membranes were probed overnight with specific primary antibodies [19] diluted in TBST: URAT1 (1:2000) and actin (1:4000), followed by secondary HRP-conjugated goat anti-rabbit IgG (Immunoglobulin G, 1:3000) antibody for 30 min. Eventually, the membranes were mixed with ECL (Enhanced Chemiluminescence, Servicebio Co., Wuhan, China) and exposed to X-ray film.

### 4.10. Molecular Docking

Initially, the crystal structure of the XOD (PDB ID: 1FIQ) was downloaded from the protein data bank [37]. Then, Protein Preparation Wizard module was applied for the preparation of protein structures, as follows: assigning bond orders, adding hydrogens, minimizing with the Optimized Potential for Liquid Simulations (OPLS_2005) force field, and then assigning protonation states. Wat176 and Wat196 in the active site were deserved [38]. Following the above, the structure underwent restrained minimization in vacuum. The minimization was carried out with the Impact Refinement module, using the OPLS-2005 force field, and terminated when the root-mean-square deviation (RMSD) reached a cutoff of 0.30 Å. The DHAP and allopurinol were minimized by exploiting LigPrep module to generate the proper ionization, tautomers, stereochemistries, and conformations, and they finally produced 255 conformations. Partial atomic charges were calculated using Merck Molecular Force Field (MMFFs) force field. The scaling factor for protein van der Waals radii was set as 0.8 Å in the receptor grid generation. The initial ligand in the active site was used as the centroid to generate the grid files for the following docking process. The default grid size was adopted from the Glide program. No constraints were applied for all the docking studies. Glide docking was performed using the Glide extra-precision (XP) mode with default protocols, and each compound was treated as flexible. Meanwhile, allopurinol, an XOD inhibitor reported in the literature, was used as a positive control. The whole process of molecular docking was completed by Schrodinger software (Maestro, Schrodinger, LLC, New York, NY, USA, 2015).

## 5. Conclusions

In summary, we report the hypouricemic effect of DHAP, a constituent screened from *G. applanatum* in silico. Its hypouricemic effect was mediated by decreasing XOD activities, along with its upregulation of OAT1 and downregulation of GLUT9, URAT1, and CNT2. Also, it showed some anti-inflammatory actions and little toxicity. Due to its inhibition against XOD, we chose XOD as a target for molecular docking studies, and revealed that three hydrogen bonds may play key roles in its orientation and binding to XOD.

## Figures and Tables

**Table 1:** Docking results of DHAP and allopurinol to XOD.

Ligands	Docking Score	XP Gscore	Glide Emodel
DHAP	−5.366	−5.426	−35.140
Allopurinol	−6.170	−6.214	−42.933

**Table 2:** PCR primer sequences and protocols.

Description	GenBank	Primer Name	Primer Sequences (5′-3′)	Product Size (bp)	T_m_ (°C)	Thermal Cycles
GAPDH ^a^	NM_008084.2	M-GAPDH-S	AGGAGCGAGACCCCACTAACA	247	60	40
		M-GAPDH-A	AGGGGGGCTAAGCAGTTGGT		60	40
GLUT9 ^b^	NM_001012363.2	M-SLC2A9-S	GATGCTCATTGTGGGACGGTT	241	60	40
		M-SLC2A9-A	CTGGACCAAGGCAGGGACAA		60	40
URAT1 ^c^	NM_009203.3	M-SLC22A12-S	CGCTTCCGACAACCTCAATG	254	60	40
		M-SLC22A12-A	CTTCTGCGCCCAAACCTATCT		60	40
OAT1 ^d^	NM_008766.3	M-SLC22A6-S	GCCTTGATGGCTGGGTCTATG	287	60	40
		M-SLC22A6-A	AGCCAAAGACATGCCCGAGA		60	40
CNT2 ^e^	NM_172980.2	M-CNT2-S	TTGGGCAAAGCGGGTGTT	135	60	40
		M-CNT2-A	CAGGCAAAGAGGATGAGGATGA		60	40

^a^ glyceraldehyde-3-phosphate dehydrogenase; ^b^ glucose tansporter 9; ^c^ uric acid transporter 1; ^d^ organic anion transporter 1; ^e^ concentrated nucleoside transporter 2.

## Abbreviations

MSU	monosodium urate
SUA	serum uric acid
HUA	hyperuricemia
XOD	xanthine oxidase
GLUT9	glucose transporter 9
OAT1	organic anion transporter 1
DHAP	2,5-dihydroxyacetophenone
BUN	blood urea nitrogen
URAT1	uric acid transporter 1
CNT2	gastrointestinal concentrative nucleoside transporter 2
GAPDH	glyceraldehyde 3-phosphate dehydrogenase
PO	potassium oxonate
HX	hypoxanthine
